# CD68+ cell count, early evaluation with PET and plasma TARC levels predict response in Hodgkin lymphoma

**DOI:** 10.1002/cam4.585

**Published:** 2016-01-13

**Authors:** Annarosa Cuccaro, Salvatore Annunziata, Elisa Cupelli, Maurizio Martini, Maria L. Calcagni, Vittoria Rufini, Manuela Giachelia, Francesca Bartolomei, Eugenio Galli, Francesco D'Alò, Maria T. Voso, Giuseppe Leone, Alessandro Giordano, Luigi M. Larocca, Stefan Hohaus

**Affiliations:** ^1^ Institute of HematologyCatholic University of the Sacred HeartRomeItaly; ^2^ Institute of Nuclear MedicineCatholic University of the Sacred HeartRomeItaly; ^3^ Institute of PathologyCatholic University of the Sacred HeartRomeItaly; ^4^ Department of Biomedicine and PreventionUniversita' Tor VergataRomeItaly

**Keywords:** CD68+ tumor‐infiltrating macrophages, Hodgkin lymphoma, interim PET, prognosis, TARC

## Abstract

Early response evaluation with [^18^F]fluordeoxyglucose (FDG) positron emission tomography after 2 cycles of chemotherapy (interim PET) has been indicated as the strongest predictor for outcome in classical Hodgkin lymphoma (HL). We studied the prognostic role of the number of tumor‐infiltrating CD68+ cells and of the plasma levels of TARC (thymus and activation‐regulated chemokine) in the context of interim PET in 102 patients with classical HL treated with Adriamycin, Bleomycin, Vinblastine, Dacarbazine (ABVD). After 2 ABVD cycles, interim PET according to Deauville criteria was negative (score 0–3) in 85 patients and positive (score 4–5) in 15 patients (2 patients technically not evaluable). TARC levels were elevated in 89% of patients at diagnosis, and decreased after 2 cycles in 82% of patients. Persistently elevated TARC levels in 18% of patients were significantly associated with a positive PET result (*P* = 0.007). Strong predictors for progression‐free survival (PFS) were a negative interim PET (85% vs. 28%, *P* < 0.0001) and CD68+ cell counts <5% (89% vs. 67%, *P* = 0.006), while TARC levels at diagnosis and at interim evaluation had no prognostic role. In multivariate analysis, interim PET, CD68+ cell counts and presence of B‐symptoms were independently associated with PFS. We conclude that although TARC levels are a biomarker for early response evaluation, they cannot substitute for interim PET as outcome predictor in HL. The evaluation of CD68 counts and B‐symptoms at diagnosis may help to identify low‐risk patients regardless positive interim PET.

## Introduction

The great majority of patients with Hodgkin lymphoma (HL) can be cured with chemotherapy or a combination of chemo and radiotherapy. There is, however, a proportion of patients, in particular those presenting with advanced stage disease, who will succumb to the disease 1. Balancing the aggressiveness of treatment between disease control and risk of short‐ and long‐term toxicity remains a challenge for treatment decisions in HL 2. Aggressive treatment of advanced stage disease using the BEACOPP regimen has certainly improved disease‐free survival, at the cost of infertility and risk of secondary organ damage and neoplasias 3, 4, 5, 6, 7. Classical clinical and laboratory risk factors at diagnosis appear to be of little help for treatment decisions in patients with advanced HL 8, 9.

In 2006, Gallamini et al. and Hutchings et al. reported that PET examination with [^18^F]fluorodeoxyglucose (FDG) after 2 cycles of standard chemotherapy, later on termed “interim‐positron emission tomography (PET)”, discriminates PET‐negative patients with a very high probability of disease control with the standard chemotherapy regimen Adriamycin, Bleomycin, Vinblastine, Dacarbazine (ABVD), from PET‐positive patients where standard therapy is most likely to fail 10, 11. These data were confirmed by several studies on patient groups with limited or advanced stage disease treated with ABVD, while the prognostic value of interim PET for patients treated with BEACOPP is not well established 12, 13, 14, 15. During a consensus meeting at Deauville in France, criteria have been standardized to evaluate interim PET, by using a 5‐point scale 16. The 5‐point scale uses uptakes by the mediastinal blood flow and the liver to quantify residual uptake in a visual evaluation. The Deauville criteria are now widely considered as the most appropriate evaluation method for interim PET 17. A PET‐guided treatment approach allows the early identification of interim PET‐positive patients, with insufficient response to standard treatment, as candidates for intensive, although potentially more toxic, treatments 18. This approach is currently evaluated in prospective studies. A drawback of the PET‐guided approach is that patients with poor prognostic features are only identified after 2 months of treatment, significantly delaying intensive treatment choices. In addition, there is a small, but consistent proportion of interim PET‐negative patients who will progress or relapse, with a progression‐free survival (PFS) around 80–85% as indicated by recent preliminary data 19. This leaves room for other potential prognosticators in addition to interim PET. For patients with refractory/relapsed HL, Moskowitz et al. showed that involvement of extranodal sites and a positive PET result pre‐high‐dose therapy were independent risk factors 20.

A particular feature of HL is that the neoplastic cells vitally depend on the supporting microenvironment. The cellular composition of the microenvironment impacts prognosis in HL. In 2010, a gene expression study by Steidl et al. pointed to the prominent role of tumor‐infiltrating macrophages in HL lymphnode biopsies 21. Over 5% tumor‐infiltrating macrophages identified by immunohistochemical staining for the CD68 antigen pick out patients at higher risk for PFS. The number of CD68+ macrophages outperformed the international prognostic score (IPS) in multivariate analysis. These data have been confirmed by several groups, including ours 22, 23, 24, 25, 26, 27, 28, 29. CD68+ cell counts appear as the most reproducible and simple prognostic marker reflecting tumor biology and is currently available, using routine diagnostic methods.

Another common feature of the tumor microenvironment in HL is the overrepresentation of tolerogenic T‐cell populations, that include T helper 2 (TH2) cells and regulatory T cells (Treg). These cells create a favorable immunological environment for the survival and proliferation of HRS cells. The chemokine thymus and activation‐regulated chemokine (TARC), also termed CCL17, engages the chemokine receptor CCR4 expressed on regulatory T and TH2 cells, recruiting these cells into HL lesions. TARC is highly expressed by HRS cells, secreted into the serum, and can be detected at high levels at HL diagnosis 30, 31, 32. Recent data suggest that early changes in TARC levels during chemotherapy may be a biomarker for response evaluation 33, 34.

We studied whether microenvironment CD68+ cell counts and TARC levels at HL diagnosis and following 2 cycles of ABVD add prognostic information to interim PET.

## Materials and Methods

### Patient characteristics

Our analysis included 102 patients (median age 38 years, range 15–74 years; 47 females and 55 males), diagnosed with classical HL and treated between February 2007 and January 2014 at the Department of Hematology of the Catholic University in Rome. Patient characteristics, including the IPS score 35 are detailed in Table 1. All patients received chemotherapy according to the ABVD protocol. Patients with limited stage disease received 3 or 4 ABVD cycles, according to the presence of other risk factors as defined by the EORTC 36, followed by involved‐field radiotherapy. Patients with advanced stage disease received 6 cycles of ABVD. Only 3 patients with advanced stage disease, with a positive interim PET result (score 4–5 according to Deauville criteria) after 2 cycles of ABVD, were switched to BEACOPP (6 cycles of dose‐escalated BEACOPP). Two of them did not achieve metabolic remission after the second‐line treatment. Radiotherapy was included for consolidation in patients with a limited‐stage disease and initial bulky disease. Informed consent was obtained from patients according to institutional guidelines. The study has been approved by the Institutional review board.

**Table 1:** Patient characteristics and interim PET

Patient characteristics	Patients	Interim PET		*P* 1
		Negative	Positive	
Number	102	85	15	
Age				
Median, range (year)	38 (15–74)	38 (15–74)	44 (20–72)	
>45 years	29 (29%)	23 (27%)	6 (40%)	0.4
Gender				
Male	55 (54%)	44 (52%)	11 (73%)	0.2
Histologic subtype				
NS	86 (84%)	71 (84%)	13 (86%)	1.0
NS 1	45 (44%)	38 (45%)	7 (47%)	
NS 2	27 (26%)	21 (25%)	4 (27%)	
MC	4 (4%)	3 (4%)	1 (7%)	
LR	2 (2%)	2 (2%)	0 (0%)	
NOS	10 (10%)	9 (10%)	1 (7%)	
Stage				
Advanced (IIB‐/IV)	49 (48%)	36 (42%)	13 (87%)	**0.002**
B‐symptoms				
Yes	38 (37%)	28 (33%)	10 (67%)	**0.02**
Bulk >5 cm				
Yes	54 (53%)	44 (52%)	9 (60%)	0.6
IPS score				
≥2	25 (25%)	15 (18%)	10 (67%)	**0.001**

NS, nodular sclerosis; NS1, nodular sclerosis type 1 according to BNLI criteria; NS2, nodular sclerosis type 2 according to BNLI criteria; MC, mixed cellularity; LR, lymphocyte‐rich; NOS: not otherwise specified; IPS, international prognostic score.^1^

*P*‐value of Fisher's exact test. Wilcoxon ranked sum test was used for comparison of median age. Two patients were excluded from the analysis, as interim PET was scored not evaluable due to unspecific FDG accumulation. Significant p‐values are shown in bold.

### Immunohistochemical analysis

Immunohistochemical analysis for CD68 was performed on 3 *μ*m tissue slides, using the antihuman mouse monoclonal antibody CD68 (1:100, clone PGM‐1; Dako, High Glostrup, Denmark) after proteolytic treatment (pronase 0.05% in tris buffer pH 7.6) for 10 min at room temperature. Immunodetection was performed using an avidin–biotin–peroxidase complex solution (ScyTek, Logan, UT), 3,39‐diaminobenzidine as the chromogen, and Mayer hematoxylin as the counterstain. We used the immunohistochemical score proposed by Steidl et al. with a cut‐off at 5% CD68‐positive cells 21.

### ELISA for plasma TARC levels

Plasma samples were collected prior to treatment start and at interim PET, and stored at −70°C. TARC levels were determined using a sandwich enzyme‐linked immunoassay, according to the manufacturer's instructions (Human CCL‐17/TARC DuoSet, R&D Systems, Inc., Minneapolis, MN). A group of 63 healthy individuals (29 males, 34 females; median age 33 years) was used as control group.

### Interim PET

PET‐CT studies were performed using an integrated PET‐CT device (GEMINI GXL distributed by Philips Medical System or BIOGRAPH distributed by Siemens. PET‐CT images were evaluated by two independent nuclear medicine physicians, using a dedicated fusion and display software (SYNTEGRA by Philips, Milan, Italy or SYNGO.VIA by Siemens, Milan, Italy).

Interim PET was performed after the second ABVD course, few days before the third course. The criteria for PET‐2 interpretation were based on visual assessment of FDG uptake, and scored for intensity of FDG uptake according to the Deauville 5‐point scoring system 16, 17. Interim PET scans with a score of 4 that equals a FDG uptake that moderately exceeds the FDG uptake in the liver, and 5 (markedly increased uptake > liver and/or new lesions related to lymphoma) were considered positive.

### Statistical analysis

Fisher's exact test was used to examine for differences in patient characteristics according to interim PET and the CD68+ cell count. Wilcoxon signed rank test was used for two‐sample comparisons of TARC plasma levels, as between patient and control groups, or according to dichotomized patient characteristics. The primary survival end point was PFS, with progression during treatment, lack of complete remission at the end of first‐line treatment, relapse, and death from any cause counted as adverse events. A positive interim PET result in the absence of progression that lead to change in therapy from ABVD to BEACOPP in 3 patients was not counted as event. Survival curves were estimated using the Kaplan–Meier product limit method. Log‐rank tests were used to analyze for differences in PFS. Hazard ratios and 95% confidence intervals were adjusted for multiple prognostic factors using the Cox proportional hazards model. All parameters that resulted significant (*P* < 0.05) in the univariate analysis were included into the multivariate analysis. These factors were: interim PET result, stage of disease, presence of B‐symptoms, IPS score, and CD68 count. In order to optimize the prognostic model, we performed a stepwise model selection using the Akaike information criterion (AIC). Computations were performed using the Stata 10.0 software (Stata Corp., College Station, TX).

## Results

### Interim PET

Interim PET‐CT scans following 2 ABVD cycles were scored according to the Deauville scoring system in 102 patients with classical HL. In 2 patients, significant FDG accumulation within brown fat tissue did not allow to discriminate for metabolic activity in sites of previous disease. The interim PET scan was scored negative (Deauville score 0–3), in 85 patients (83%), while it was positive in 15 patients (15%). Looking at patients' characteristics, there was a significant association between interim PET‐positivity, advanced stage of disease (*P* = 0.002), presence of B‐symptoms at diagnosis (*P* = 0.02) and an IPS score >2 (*P* = 0.001) (Table 1).

### TARC levels at diagnosis and interim PET

**Figure 1:**
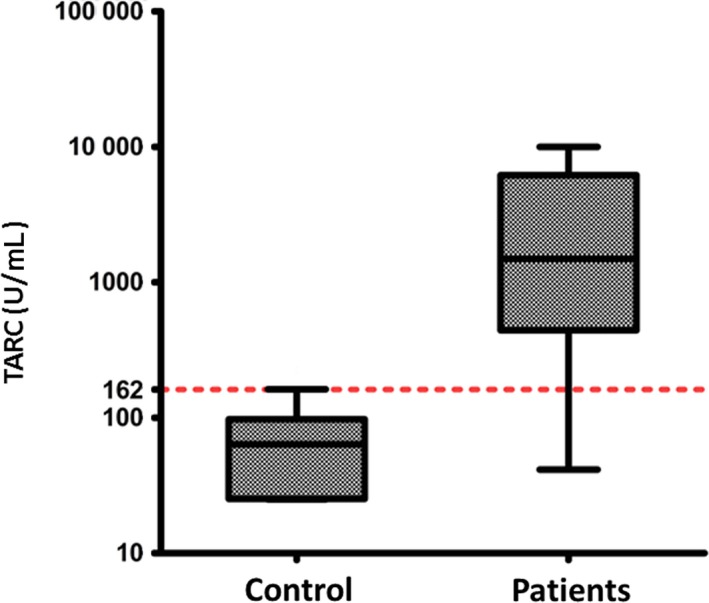
Thymus and activation‐regulated chemokine (TARC) Plasma levels are increased in Hodgkin lymphoma (HL) patients at diagnosis than in controls. TARC levels were significantly higher in HL patients (*n* = 80, median 162 U/mL) when compared to controls (*n* = 63, median 64 U/mL) (*P* < 0.001). The upper border of the box indicates the 75th percentile, while the lower border indicates the 25th percentile, and the horizontal line in the box the median. The vertical lines are the whiskers indicating the maximum and minimum values. TARC levels in controls were used to define the upper normal value (dashed line, 162 U/mL).

**Figure 2:**
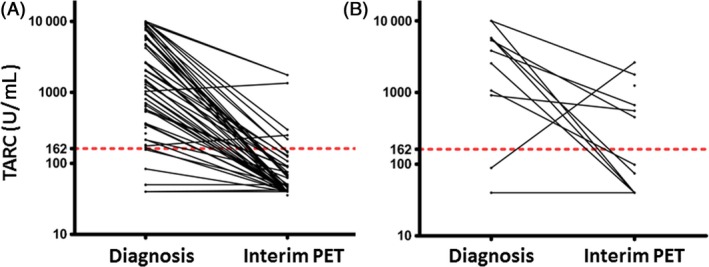
Elevated Plasma levels of thymus and activation‐regulated chemokine (TARC) at diagnosis predicted interim positron emission tomography (PET) results. TARC levels were elevated at interim PET in only 6 of 51 (12%) patients with a negative interim PET (A), while they persisted elevated in 6 of 12 (50%) patients with a positive interim PET (B, *P* = 0.007). The dashed line indicates the upper normal value.

TARC levels were determined at diagnosis in 80 patients, and were significantly higher than those of controls (*P* > 0.001) (Fig. 1). TARC levels were above the normal range (upper limit 162 U/mL) at diagnosis in 89% (71/80) of patients with cHL. Significantly higher TARC levels were observed in patients younger than 45 years (*P* = 0.02), and patients with bulky disease (*P* = 0.02) (Table 2). Plasma was available at the time of interim PET in 65 patients. TARC levels decreased at interim PET in 57 patients, but persisted elevated (>162 U/mL) in 12 patients (18%) (Fig. 2). Persistently elevated TARC levels were significantly associated with a positive PET result (*P* = 0.007) (Fig. 2).

**Table 2:** Thymus and activation‐regulated chemokine (TARC) levels according to patient characteristics

Parameter	Variable	Cases (*n* = 80)	TARC (U/mL) median	*P* 1
Age	<45 years	58	2613	**0.02**
	>45 years	22	811	
Gender	Female	40	2571	0.07
	Male	40	949	
Histologic subtype	NS	70	1885	0.1
	Others	10	661	
Stage	Limited	42	1100	0.08
	Advanced	38	2612	
B‐symptoms	No	55	1182	0.2
	Yes	25	2059	
Bulk >5 cm	No	31	942	**0.02**
	Yes	47	3829	
IPS	IPS 0–2	61	1482	0.7
	IPS 3–7	19	2554	
CD68+	<5%	30	3206	0.1
	>5%	33	924	

^1^ *P*‐value of Wilcoxon ranked sum test that was used for comparison of TARC levels according to patient characteristics.

Significant p‐values are shown in bold.

### Association of the number of CD68+ macrophages with patient characteristics and interim PET results

Tumor biopsies of 79 patients at the time of HL diagnosis were stained for the CD68 antigen and scored using the immunohistochemical system proposed by Steidl et al. 21. Over 5% CD68 positive cells were counted in 39/79 (49%) patients. CD68+ cell counts >5% were frequent in patients aged over 45 years (17/22, 77%), when compared to younger patients (24/59, 41%) (*P* = 0.005). There were no other associations between CD68+ cell count and patient characteristics as listed in Table 1.

We observed a direct correlation between interim PET score and CD68 cell counts in the tumor biopsy. When grouping patients according to the consensus cut for PET‐positivity, which is score 4 16, the association between a positive interim PET and higher CD68 counts was not significant (8/39, 21% vs. 4/40, 10%; *P* = 0.2). On the other hand, patients with interim PET score 1 or 2 according to the Deauville system frequently had less than 5% CD68+ counts, while patients scored 3–5 had significantly higher CD68+ cell counts at diagnosis (*P* < 0.05) (Table 3).

**Table 3:** CD68+ cell count and interim positron emission tomography (PET)

Interim PET (Deauville score)	CD68		Total no. of patients
	<5%	>5%	
1	**18**	15	33
2	**17**	9	26
3	1	**7**	8
4	4	**7**	11
5	0	**1**	1
Total	40	39	79

*P* < 0.05, Fisher's exact test. Patients with CD68 counts <5% had lower Deauville scores at interim PET when compared to patients with CD68 counts >5%.

Significant p‐values are shown in bold

### Associations of CD68+ cell count and interim PET with outcome

**Figure 3:**
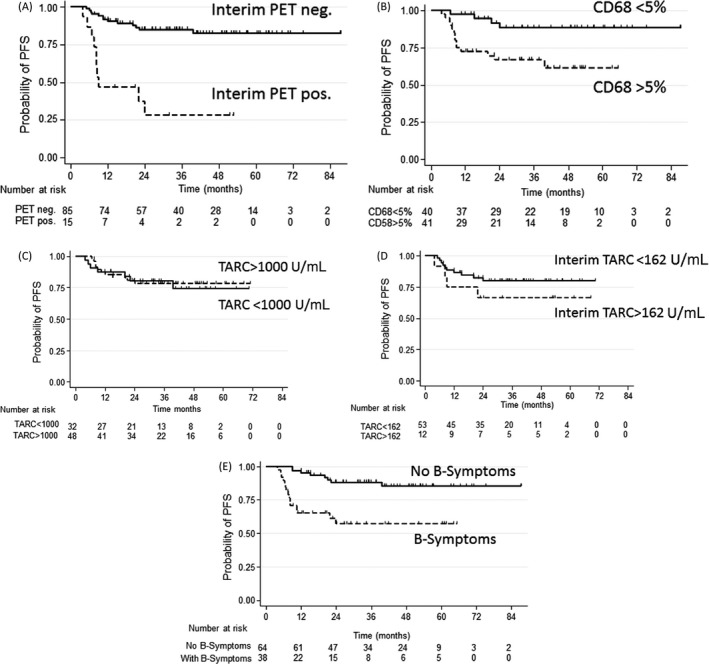
Progression‐free Survival according to interim [^18^F]fluordeoxyglucose (FDG)‐positron emission tomography (PET)/CT scan, CD68+ cell counts and thymus and activation‐regulated chemokine (TARC) levels at diagnosis and at interim PET. Patients with an interim PET score of 1–3 were considered PET‐negative (*n* = 85), and patients with a score of 4–5 were scored PET‐positive (*n* = 15). After a median observation time of 32 months, the probability of progression‐free survival (PFS) was 85% (95% CI: 75–91%) for PET‐negative patients, while it was only 28% (95% CI: 8–53%) for PET‐positive patients. The difference was highly statistically significant (*P* < 0.0001). (A) Patients with CD68+ cell counts <5% (*n* = 40) had a significantly higher probability of PFS at 32 months (89%, 95% CI: 72–96%), than patients with CD68+ cell count >5% (*n* = 39) (67%, 95% CI: 50–79%; *P* = 0.006). (B) The PFS probability was similar for patients with TARC levels below or over >1000 U/mL at initial diagnosis (*n* = 32 and *n* = 48, respectively) (C) Petients with increased TARC levels (>162 U/mL, *n* = 12) at interim PET had a PFS (PFS: 67%, 95% CI: 34–86%), similar to patients with TARC levels <162 U/mL (*n* = 53, PFS: 80%, 95% CI: 66–89%, *P* = 0.3). (D) Patients with B‐symptoms (*n* = 38) had a significant inferior probability of PFS (57%, 95% CI: 39–72%), when compared to patients without B‐symptoms (*n* = 64) (88%, 95% CI:77–94%) (*P* = 0.002).

At a median follow‐up of 32 months (range 4–88 months), 23 of 102 patients had disease progression, which translated into a 77% probability of PFS (95% CI, 67–84%). The univariate analysis showed that positive interim PET was associated with significantly worse PFS (*P* < 0.0001) (Fig. 3A), as was the number of CD68+ macrophages (*P* = 0.006) (Fig. 3B), advanced stage of disease (*P* = 0.008), presence of B‐symptoms (*P* = 0.0002, Fig. 3C) and IPS score >2 (*P* = 0.009, data not shown). TARC levels at diagnosis and at interim PET had no prognostic significance (Fig. 3D and E). We also analyzed the prognostic relevance of interim TARC levels in addition to interim PET results. Interim TARC levels did not add any prognostic information to interim PET (*P* = 0.4 for interim PET negative patients, *P* = 0.5 for interim PET positive patients).

The multivariate Cox proportional hazard regression analysis showed that interim PET, number of CD68+ cells and the presence of B‐symptoms were independent prognostic factors (*P* = 0.001, *P* = 0.01, and *P* = 0.04, respectively), while the IPS score and stage of disease did not play a significant role (Table 4). A stepwise model selection using the AIC confirmed a multivariate model including the interim PET, number of CD68+ cells, and presence of B‐symptoms.

**Table 4:** Multivariate Cox analysis of progression‐free survival of 79 patients with HL, treated with ABVD

Variable		Hazard ratio	95% CI	P*
PET	Positive versus negative	7.5	2.3–24.7	0.001
CD68	>5% versus <5%	4.3	1.4–13.5	**0.01**
Stage	IIB‐IV versus I‐IIA	0.8	0.2–3.3	0.8
B‐symptoms	Yes versus no	4.4	1.1–18.6	**0.04**
IPS score	>2 versus 0–2	0.8	0.3–2.5	0.8

Significant p‐values are shown in bold.

**Figure 4:**
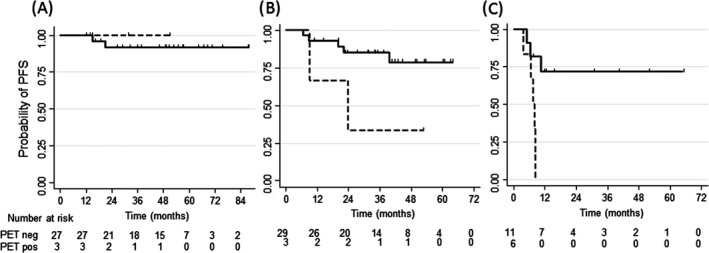
Progression‐free survival according to interim positron emission tomography (PET) scan in patients according to CD68+ cell count and B‐symptoms. PFS curves are shown for patients with CD68+ <5% and no B‐Symptoms (A), CD68 > 5% or B‐symptoms (B), and CD68% >5 and B‐symptoms (C). The continuous line indicates patients with negative interim PET, the dashed line patients with a positive interim PET. The survival difference is significant in patients with both CD68 counts >5% and B‐symptoms (*P* = 0.003), while there is a trend for significance in the group with either CD68 count>5% or B‐symptoms (*P* = 0.06).

Integrating the three significant parameters, we were able to identify patient groups characterized by significantly different prognosis. Patients (*n* = 30) with <5% CD68+ cell counts, without B‐symptoms had a 92% (95% CI: 72–98%) probability of PFS, independent from interim PET scan result (Fig. 4A). Patients with both B‐symptoms and CD68+ cell counts >5% (*n* = 17) had a poor prognosis (PFS of 45%; 95% CI: 20–66%). In this group, a negative interim PET identified a still favorable prognostic group, compared to patients with a positive PET (PFS: 72% vs. 0% for patients with a positive interim PET, *P* = 0.003) (Fig. 4C). Patients with either B‐symptoms or CD68+ cell counts >5% (*n* = 31) and a negative interim PET still had a trend for better outcome (85% vs. 33% at 32 months, *P* = 0.06) (Fig. 4B).

## Discussion

We report that the number of CD68+ tumor‐infiltrating macrophages and presence of B‐symptoms are prognostic markers in ABVD‐treated HL also in the era of interim PET, while changes in TARC levels do not add prognostic information. More importantly, this is the first study showing that the integration of interim [^18^F]‐FDG‐PET/CT scan results after 2 chemotherapy cycles with presence of B‐symptoms and CD68+ cell counts at diagnosis, improves risk‐stratification of patients with HL. This additional information may help to identify a group of low‐risk patients for whom interim PET is not predictive, and a high‐risk patients group who are still at risk also when PET is negative. In our study, the interim PET evaluation was performed according to the recommended 5‐point Deauville scale 17. PET results in our patients are in line with other recent reports on 84% PFS of interim PET‐negative patients 19. However, these figures are lower than initial studies, who reported more than 90% PFS in interim PET‐negative patients 10, 11, 19.

We found that TARC plasma levels were elevated at diagnosis in the vast majority of patients with classical HL, and turned to normal following 2 cycles of ABVD. Persistently elevated TARC levels were associated with a higher risk of a positive interim PET. This is in line with two recent studies suggesting that early changes of TARC may be a useful blood biomarker 33, 34. However, in our study absolute TARC levels or their changes did not add prognostic information to interim PET. Studied with larger patient numbers and events are required to address this issue.

Macrophage count was higher than 5% in about half of the patients, and was associated to patients' age over 45 years. This is in line with our previous observation, indicating that the number of CD68+ cells is higher in EBV‐associated HL that is typically more frequent in older patients 22. Patients with >5% CD68+ cell counts were likely to have a higher (≥3) Deauville score at interim PET. A Deauville score of 3 defines a residual FDG uptake higher than the mediastinal blood flow, but not exceeding activity in the liver. Potential associations between the CD68 count in the microenvironment and PET results in HL have been recently addressed, with conflicting results. Touati et al. 28, reported that the frequency of CD68+ cells correlates to interim‐PET results. In contrast, Agur et al. 29 reported that CD68 counts correlate to the initial tumor mass and residual tumor size, but not to interim PET result and PFS. We think that homogenous treatment, standardized scoring of interim PET according to Deauville criteria, and CD68 evaluation by an expert hematopathologist, eliminating inter‐observer variability are key issues of quality in our study.

The direct correlation between number of CD68+ macrophages and Deauville score at interim PET may indicate that lymphomas with higher initial macrophage content are at higher probability of treatment resistance, or that persisting macrophages contribute to residual accumulation of [^18^F]‐FDG. On the other hand, one could also speculate that persisting macrophages stimulate metabolic activity of HRS cells.

We found that the presence of B‐symptoms is an independent prognosticator in the multivariate analysis. B‐symptoms are associated with a variety of other laboratory abnormalities and patient characteristics, in particular advanced‐stage disease, and therefore have been often removed in multivariate analyses models, as in the IPS 35. B‐symptoms are due to the production of pro‐inflammatory cytokines by the Hodgkin tumor tissue, in particular IL‐1, TNF‐alpha, and IL‐6, which are detected at increased levels in peripheral blood 37. We found that TARC levels are not predictive of outcome. On the other hand, cytokine models including IL‐6, sCD30 and TNFR1 levels was more predictive than the standard clinical score 37. It will be of great interest to explore whether in the multivariate analysis including CD68+ cell counts and interim PET, the cytokine score could beat clinical characteristics as B‐symptoms.

Our study shows that histological (CD68 counts) and clinical characteristics at diagnosis provide prognostic information already at treatment start, and not only after 2 cycles of ABVD. Patients with low CD68+ cell counts and without B‐symptoms may have a favorable outcome, despite a positive interim PET scan. On the other hand, patients with high CD68+ cell counts and B‐symptoms are at high risk, and treatment results may be poor also in the presence of a negative interim PET. It remains to be determined whether this group could benefit from intensified treatment strategies, as the BEACOPP regimen, or addition of new agents, as brentuximab vedotin, right from therapy start. In advanced HL, we are still in need of markers that are not only prognostic, but predictive and can help to tailor first‐line therapeutic approaches.

In conclusion, the frequency of CD68+ macrophages at HL diagnosis remains a significant prognostic marker. Patients with a CD68 count >5% and B‐symptoms are at high‐risk for early progression. It is important to underline that the numbers of this subgroup analyses are small and have therefore to be considered explorative. Technological improvements in the near future using the nanostring technology will probably render the evaluation of the tumor‐associated macrophage count more robust and overcome some variability due to the use of different antibody clones (KP1 and PGM1), varying thresholds and inter‐observer differences 38. Moreover, validation of our data on a potential prognostic algorithm integrating information at diagnosis and response evaluation according to interim PET‐CT may provide the basis for innovative risk‐adapted treatment protocols in HL.

## Conflicts of Interest

The authors have no conflicts of interest to declare.
